# Psychological Factors Influencing Pro-environmental Behavior in Developing Countries: Evidence From Colombian and Nicaraguan Students

**DOI:** 10.3389/fpsyg.2020.580730

**Published:** 2020-12-23

**Authors:** Manuel Francisco Díaz, Andrés Charry, Stefania Sellitti, Matteo Ruzzante, Karen Enciso, Stefan Burkart

**Affiliations:** ^1^Tropical Forages Program, International Center for Tropical Agriculture (CIAT), Cali, Colombia; ^2^Food Environment and Consumer Behavior Research Lever, International Center for Tropical Agriculture (CIAT), Cali, Colombia; ^3^NOVA School of Business and Economics, Lisbon, Portugal; ^4^Development Impact Evaluation, World Bank, Washington, DC, United States

**Keywords:** awareness of sustainability, education, psychological adaptation, environmental attitude, policy support

## Abstract

Identifying the determinants of human behavior is useful to adjust interventions and lead the civil society toward a stronger commitment to climate change (CC) mitigation and adaptation objectives, achieving greater support for successfully implementing environmental policies. Existing research has largely focused on case studies of pro-environmental behaviors (PEBs) in developed economies but there is very little evidence for developing countries. This study provides estimations of the effect of internal factors, such as sociodemographic variables, and four psychological dimensions (CC knowledge, environmental attitudes, self-efficacy, and trust in sources of environmental information) on PEBs. Data were obtained through a survey applied with future decision makers – university students – from Colombia (*n* = 4,769) and Nicaragua (*n* = 2,354). Indices were generated for PEBs and the psychological dimensions using *z*-scores and Principal Component Analysis (PCA). Partial correlations were evaluated through the Ordinary Least Squares (OLS) method. Our results suggest that, in order to reach the planned emission reduction targets, policy approaches should more strongly focus on educating and motivating citizens and prepare them for contributing to the environmental cause, as well as provide individual solutions to combat CC, rather than providing only information on its causes and consequences.

## Introduction

As part of the commitment with the Sustainable Development Goals and the Paris Agreements, developing countries have been increasing their responses to climate change (CC), especially since evidence suggests that the impacts of CC would have larger impacts in the global south, strengthening structural inequalities and leading to a vicious circle (Burke et al., 2015; United Nations, Department of Economic and Social Affairs, 2016). This panorama has led to a change in the development model of nations with a deliberate direction toward sustainability (Bárcena et al., 2018; Intergovernmental Panel on Climate Change, 2018), resulting in programs and strategies for CC mitigation and adaptation, which demand context specific approaches. However, the success of such approaches depends largely on the social norms, preferences, beliefs and values of the targeted individuals (Adger et al., 2009). Factors such as public awareness and knowledge of CC, attitudes and opinions regarding environmental problems, and knowledge about appropriate behaviors determine the public support or opposition of environmental or CC policies, strategies, and initiatives (Arcury, 1990; Leiserowitz, 2006; Lorenzoni et al., 2007; Howe et al., 2015). Lee et al. (2015) argue that some countries are more advanced than others in terms of executing environmental policies resulting from differences in risk perception of the targeted populations.

During the last 4 decades, important advances were made in the identification of factors influencing environmental perceptions and Pro-environmental Behaviors (PEBs). Nevertheless, most of these studies were conducted in North America, Europe, and other developed regions (Lorenzoni and Pidgeon, 2006; Cordano et al., 2010; Vignola et al., 2013; Salehi et al., 2016). While research on PEBs has been growing in Latin American countries recently (Padilla y Sotelo and Luna, 2003; Pato et al., 2005; Bertoni and López, 2010; Calixto and Herrera, 2010; Barazarte et al., 2014; Sánchez et al., 2014; González and Maldonado, 2015; Ideam et al., 2016; Pávez-Soto et al., 2016). These studies have covered different groups such as students (e.g., Tikka et al., 2000; Spellman et al., 2003; Palavecinos et al., 2016; Salehi et al., 2016), consumers (e.g., Tobler et al., 2012a,b; Yadav and Pathak, 2016), citizens with diverse political and religious positions (e.g., Arbuthnot, 1977; Tobler et al., 2012b), professors (e.g., Pe’er et al., 2007) and communities related to recycling (e.g., Sidique et al., 2010).

This study aims at enhancing the knowledge base for the Latin American context by evaluating perceptions and behavior toward CC with a large sample of university students in Colombia and Nicaragua. Knowledge and attitudes about CC, self-efficacy, and trust in different information sources are measured, and relationships within PEBs, knowledge, attitudes, and socioeconomic characteristics of the selected population are explored.

The applied approach leads to two questions: (1) Why choose this segment of the population as study group? and (2) Why identifying relationships among the variables? According to Bradley et al. (1999), university students – future scientists, legislators, consumers, and voters – will be responsible for generating solutions to environmental problems, and thus should be persuaded to adopt and pay the costs of future environmental policies. Likewise, students will have to make complex political decisions about CC mitigation and should do so from an informed perspective. Consequently, current and future educators require a better understanding of the dimensions affecting the students’ perceptions in order to develop teaching programs that contribute in a more effective way to the fight against CC (Wachholz et al., 2014). Next, identifying the relationships between PEBs and the variables that affect them provides a clearer landscape to define strategies and prioritize efforts for increasing the level of environmental awareness. Accordingly, this research allows identifying the most reliable agents in disseminating information, and provides guidance on the type of knowledge that should circulate in order to improve the efficacy of both public and private communication strategies. In addition, policy approaches will be more effective when taking into account the psychosocial context and factors that influence environmental actions (Steg and Vlek, 2009; Stern, 2011), considering that students not only increase their own contribution to CC mitigation, but also their empowerment to become change agents and influencers for other segments of the population (González and Maldonado, 2015).

## Theoretical Framework

### The Theory of Planned Behavior (TPB) as a Guiding Principle

Theory of Planned Behavior (TPB; Ajzen, 1985, 1991) is considered an extension of the Theory of Reasoned Action (TRA) (Ajzen and Fishbein, 1980), which explains behaviors under a logical framework: behavioral beliefs are supported by a favorable or unfavorable attitude about a certain behavior. Normative beliefs refer to the subjective norm and thus the social pressure associated with behavior. In this sense, actions are supported by individual attitudes, available information, and subjective norms, which are based on beliefs formed through knowledge, understood as the element that allows evaluating the consequences of actions. TPB introduces an additional element: *the control of perceived behaviors*. This element refers to the understanding of the factors that can hinder the performance of actions and the subsequent behavior derived from them (Ajzen, 1991). The theory suggests that people are much more likely to adopt a certain behavior when they feel able to perform it successfully, a dimension also affected by self-efficacy (Bandura, 1977). This concept refers to “*people’s beliefs about their capabilities to exercise control over their own level of functioning and over events that affect their lives*” (Bandura, 1991, p. 257).

While TRA and TPB models do not include sociodemographic variables, the authors do not deny their importance. Rather the opposite: they argue that any external variable can influence the intention – and indirectly, the actual behavior – if it influences the attitudinal and/or the normative component. Although some studies have not taken into account sociodemographic variables to relate PEBs with the theories presented above (e.g., Bang et al., 2000; Mishra et al., 2014; Paul et al., 2016; Beckage et al., 2018), others did (e.g., Goldenhar and Connell, 1993; Kim et al., 2013; Paço and Lavrador, 2017). The latter shows that the relationship between knowledge, attitudes, behaviors, and external variables differ among contexts change over time and are perceived differently from one culture to another. This highlights the importance of combining sociodemographic and cognitive factors to study PEBs in context-specific cases.

Various studies have suggested that the general framework of TPB could be enriched and broadened by adding new constructs or altering the pattern of variables contemplated in the TRA and TPB (e.g., Perugini and Bagozzi, 2001; Moons and De Pelsmacker, 2015; Yadav and Pathak, 2016; Wan et al., 2017). This is common practice: for example, in a meta-analysis on the application of the TPB to examine environmental behaviors, Yuriev et al. (2020) found that 72% of the analyzed studies used an extended version of the TPB. By including these constructs, these studies added factors that increase the predicting power of the model and may account for observed differences between groups, as they take into account specific contextual and idiosyncratic factors that can influence behavior.

Similar to these approaches, the present study is inspired by but extends on the TPB, to the extent that it involves dimensions different to the ones described above, such as sociodemographic characteristics, knowledge of CC, and self-efficacy. Additionally, the dimension “*trust in sources of environmental information*” was included with the aim of exploring both the individual and broader explanatory factors and thus improve the predictive power of the framework and identify sources of variation between such stated behaviors. The next section presents the selected dimensions and their importance for the present study.

### Explanatory Variables

Similar studies have addressed topics such as energy saving (e.g., Sapci and Considine, 2014), recycling (e.g., Goldenhar and Connell, 1993; Sidique et al., 2010; Paço and Lavrador, 2017) or the willingness to pay for environmentally friendly products (e.g., Furlow and Knott, 2014; Paul et al., 2016; Bedard and Tolmie, 2018), using a broad set of possible explanatory variables. These range from psychological dimensions (Arbuthnot, 1977; Helm et al., 2018), to the orientation of messages (Gifford and Comeau, 2011), geographical variation (Howe et al., 2015), or a combination of both (Zhang et al., 2018).

For this study, socioeconomic characteristics and four dimensions were determined for their potential explanatory relationship with PEBs. PEBs can be understood in two ways: first, as behavior that “*harms the environment as little as possible, or even benefits the environment*” (Steg and Vlek, 2009, p. 309) and second, as behavior “*that is undertaken with the intention to change (normally, to benefit) the environment*” (Stern, 2000, p. 408). In order to simultaneously identify factors that influence both future intentions and current PEBs undertaken by the population of interest, this construct includes 14 statements referring to both declared behaviors and intentions to conduct PEBs.

#### Sociodemographic Variables

##### Gender

Women and men do not experience CC in the same way. Literature shows that women, particularly in rural areas, present greater concerns about CC since they carry out activities such as raising children, or planting and harvesting, which depend largely on both natural resources and a healthy environment (Blocker and Eckberg, 1989; Davidson and Freudenberg, 1996; Food and Agriculture Organization of the United Nations, 2015; Vicente-Molina et al., 2018). In that sense, women are strongly affected by changes in the environment and show to be more committed to mitigating actions (Bord et al., 1998; O’Connor et al., 1999; Gifford and Comeau, 2011; Perez et al., 2015; Ideam et al., 2016; Palavecinos et al., 2016; Paço and Lavrador, 2017). Among younger people, women have also shown better environmental attitudes and knowledge, are more concerned about environmental problems, and are more involved in CC mitigation actions (e.g., Freudenburg and Davidson, 2007; McCright, 2010).

##### Age

Children and elderly experience more aggressively the effects of CC. Both populations present higher mortality and disease rates due to hurricanes, floods, and droughts (Intergovernmental Panel on Climate Change, 2014b). Though Otto and Kaiser (2014) found that older people have better PEBs than younger individuals, the effect of age is ambiguous and appears to be affected by access to information. Furlow and Knott (2014) and Bedard and Tolmie (2018) argue on the importance of the internet and digital communications for younger generations, who tend to be better informed and more concerned about social and environmental issues. As a result, younger individuals have more tools to understand CC and consequently generate environmental actions.

##### Geography

Similar demographic and cultural characteristics tend to cluster (Leiserowitz, 2006; Motyl et al., 2014; Howe et al., 2015). Likewise, perceptions of CC exhibit geographic patterns due to differences in experiences with extreme weather events and climate variability (Akerlof et al., 2013; Howe et al., 2015). In their analysis in 89 countries, Howe et al. (2013) found that people living in places more susceptible to CC are the most concerned about the phenomenon.

##### Field of Study

Students of certain academic fields show a better understanding of CC. Several authors (e.g., Tikka et al., 2000; Spellman et al., 2003; Pe’er et al., 2007; Salehi et al., 2016) found that students from disciplines related to environmental and natural sciences possess a significantly higher level of environmental knowledge and attitudes than those from other programs.

##### Education Level and Academic Cycle

Educational achievements are the strongest predictor for environmental knowledge and understanding of CC (Polonsky et al., 2011; Lee et al., 2015). According to Meyer (2015), education can lead people to care more about general social welfare, including the external benefits of their actions. Furthermore, the time spent at university can have a positive impact on individuals, since higher education institutions tend to encourage students to incorporate principles of environmental responsibility (Kagawa, 2007; Emanuel and Adams, 2011). Spellman et al. (2003), Meyer (2016), and Paço and Lavrador (2017) have found significant differences between students of higher semesters and those who have recently started their studies. However, other authors did not find significant relationships between knowledge of CC and the academic cycle (e.g., Salehi et al., 2016).

##### Income Level/Socioeconomic Strata

People with less resources are the most affected by CC despite not being the main emitters of greenhouse gas (GHG; Mendelsohn et al., 2006; Intergovernmental Panel on Climate Change, 2014a; Hallegatte and Rozenberg, 2017; International Monetary Fund, 2017; Bárcena et al., 2018). Low-income people are often located in places more vulnerable to climatic phenomena and experience higher levels of worries and a greater sense of insecurity. They are the ones who know more about the effects of CC, but lack an adequate understanding of the causes as well as strategies for coping with the consequences (Hardoy and Pandiella, 2009), and therefore the relationship with PEBs may be ambiguous.

#### Dimensions of Reference

##### CC Knowledge

A higher knowledge about CC leads to increasing concerns about it. Consequently, informed citizens are more likely to perform actions that promote environmental protection and support related policies (Ramsey and Rickson, 1976; O’Connor et al., 1999; Bord et al., 2000; Kellstedt et al., 2008; Shi et al., 2016). However, CC is a complex phenomenon that encompasses multiple causes and a great variety of consequences. Various authors found that, in order to properly face CC, knowledge about the anthropogenic causes of CC might be more relevant than, for example, knowledge about its physical effects (Bord et al., 2000; Lee et al., 2015; Shi et al., 2016). Other studies (e.g., Tobler et al., 2012a; Salehi et al., 2016) present the need to know and differentiate the causes and consequences of CC as well as the knowledge of concrete actions to mitigate it. However, Shi et al. (2016) state that it is essential to focus studies on all dimensions since measuring the perceptions of the phenomenon transversally becomes necessary.

##### Self-Efficacy

This dimension is of special relevance for the TPB since it contributes to the determination of perceived behavioral control and thus to PEBs. Expectations such as motivation, performance, and feelings of frustration determine behavioral reactions. Some studies demonstrate the perceived efficacy of individual actions in the fight against CC, showing how deeply they influence PEBs and environmental knowledge (Heath and Gifford, 2006; Kellstedt et al., 2008). Not only that, high self-efficacy can influence the transition from easy-to-perform PEBs to those with a greater degree of difficulty (Lauren et al., 2016).

##### Trust in Sources of Environmental Information

Discourses projected by the media contribute to the evaluation and social interpretation of a problem (like CC). By this, media help transferring certain elements of science into common culture (Pinheiro and Farias, 2015). Although neither TRA nor TPB consider this dimension within their models, it is necessary to bear in mind both the representations and the social interactions through which common culture is constructed and shared (Meira-Cartea et al., 2018). Not only that, decision-making is dependent not only on the availability of information but also on the level of trust in different sources (Dietz et al., 2007). Lorenzoni et al. (2007) describe that public distrust in media constitutes an important impediment to CC adaptation. According to them, media tactics such as exaggeration, sensationalism, or partiality (in addition to contradictory frames) end up generating confusion. Gifford and Comeau (2011) found that the orientation of messages influences both the commitment toward mitigation and the intentions of the behavior. Kellstedt et al. (2008) state that trust in Non-Governmental Organizations (NGOs), media, or political institutions can facilitate or obstruct the understanding of CC. Spence and Pidgeon (2010) found that the effectiveness of the messages also varies according to the geographic location where the information circulates, with different effects on the recipient’s behavior.

##### Environmental Attitude

The inclusion of attitudes as an explanatory factor of behavior is the most adopted approach (Li et al., 2019). Authors such as Arcury (1990) and Kaiser et al. (1999) affirm that there exists a link between knowledge and PEBs, and that they are generally connected by attitudes. To measure this dimension, the present study adopts the New Environmental Paradigm (NEP) scale. Dunlap and Van Liere (1978) propose NEP to respond, not only to different development theories, but also to a new way of understanding the relationships between human beings and their environment, which translates into a radical change in attitudes. By covering different environmental issues with relative standardization, the NEP has become a conventional scale to capture this information.

## Materials and Methods

### Participants

**Figure 1:**
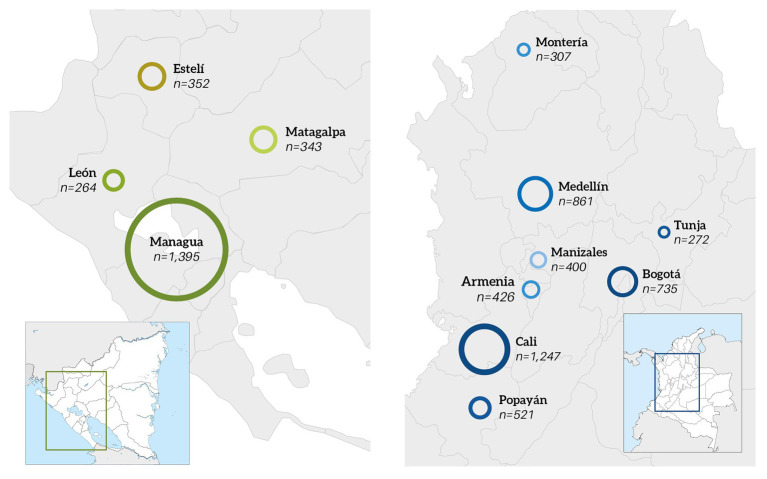
Geographical distribution of the study population for Nicaragua (left) and Colombia (right).

Higher education students from the main cities of Colombia and Nicaragua were selected as the objective population of this study. A sampling frame was developed using a list of universities from four large cities in Nicaragua and 10 in Colombia, respectively. Simple random sampling was carried out to select the universities, and the survey was then applied to students belonging to the faculties where authorization was granted. Total number of valid surveys obtained was 7,123: 2354 in Nicaragua and 4,769 in Colombia. Respondents of the city of Managua concentrated 59.2% of the Nicaraguan sample, whereas in Colombia, the cities with higher participation were Cali (26.1%), Medellín (18%) and Bogotá (15.4%; Figure 1). Table 1 shows the most relevant educational and socio-demographic characteristics of the sample.

**Table 1:** Educational and sociodemographic characteristics of the study population in Nicaragua and Colombia.

	Nicaragua (%)	Colombia (%)
	(*n* = 2,354)	(*n* = 4,769)
Age (average in years)^***^	19.6 (*σ* = 2.62)	21.3 (σ = 4)
**Gender^a^**		
Female	44.3	49.5
Male	54.4	50.1
**Income level (Nic – US$)/socioeconomic strata (Col)^a^**		
≤ US 250/1	31.5	13.9
251–500/2	35.6	31
501–750/3	7.6	33.9
751–1,000/4	3.7	10.7
1,001–1,250/5	6.7	4.9
≥1,251/6	11.7	2.1
Education level^***^		
Candidate undergraduate degree (BSc)	99.6	97.3
Candidate postgraduate degree (MSc, PhD)	0.4	2.7
Academic cycle^a^^***^		
First year	35.9	31.7
Second year	20.7	19.3
Third year	17	16.2
Fourth year	14.5	18.4
Fifth year	11.9	13.8
**Field of study**		
Agricultural sciences	44.4	32.6
Engineering	30.5	24.7
Natural sciences	1.1	11.1
Health sciences	10	2.3
Administrative sciences	7.8	7.8
Others^b^	6.2	21.5

a Not all percentages add up to 100%. Some participants did not provide all the requested information.

b Others: Humanities, Laws, Basic sciences, Arts, pedagogy and comunications.

*** *p* < 0.001.

Both the survey and methodology were approved by the International Center for Tropical Agriculture’s ethics committee. Before the administration of the survey, the students were provided with information on the objectives of the study, data privacy and management, and their rights as participants, after which an informed consent was confirmed. This consent highlighted three aspects: (1) the use of data for academic research purposes only and protection of the participant’s identity, (2) the voluntary character of participation and opportunity for withdrawal at any time, and (3) the possibility for participants to request a copy of the results after analysis.

### Instruments

In addition to sociodemographic information, data to measure different dimensions were obtained using a five-point Likert-type survey. The instrument included five modules with a series of questions and statements related to the previously described dimensions, presenting 48 statements. To verify the internal consistency of the instrument, a pilot test was conducted with 100 students from the city of Cali (Colombia) and a Cronbach’s alpha coefficient was calculated for each variable (Table 2). For data collection, Information on income was captured differently in both countries: in Nicaragua, the level of income was measured in monetary terms. In Colombia, the measurement was in accordance with the national socioeconomic stratification categories,^1^ ranging from one to six (the higher the number, the better the living conditions in the place where the respondent’s household is located), which normally is used to relate to the level of income and socioeconomic conditions of an individual. In both countries, the survey was mainly conducted at university classrooms through self-administered questionnaires. In Colombia, some students participated through an online survey, which was sent to their institutional emails by the respective university authorities.

**Table 2:** Dimensions of the survey.

Dimension	Selected references	Selected items	Total values	Cronbach’s alpha (α) pilot test
CC knowledge	Spellman et al. (2003)	16	80	0.64
Self-efficacy	Kellstedt et al. (2008)	4	20	0.62
Trust in sources of environmental information	Kellstedt et al. (2008)	6	30	0.65
Pro-environmental behaviors	Markle (2013), Paço and Lavrador (2017)	14	70	0.73
New Environmental Paradigm (NEP)	Dunlap and Van Liere (1978)	8	40	0.71

### Data Analysis

#### Exploration of Data

**Figure 2:**
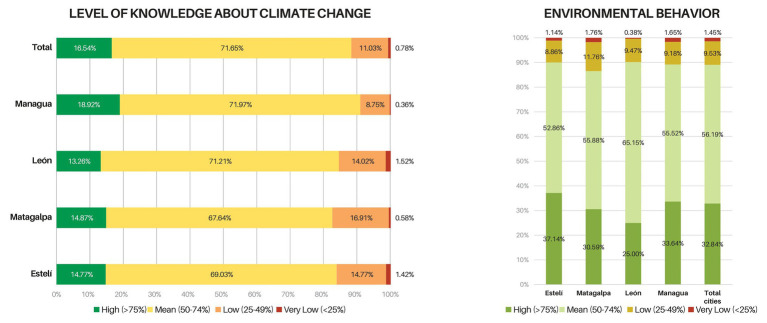
Knowledge about climate change (left) and PEBs (right) by cities in Nicaragua.

**Figure 3:**
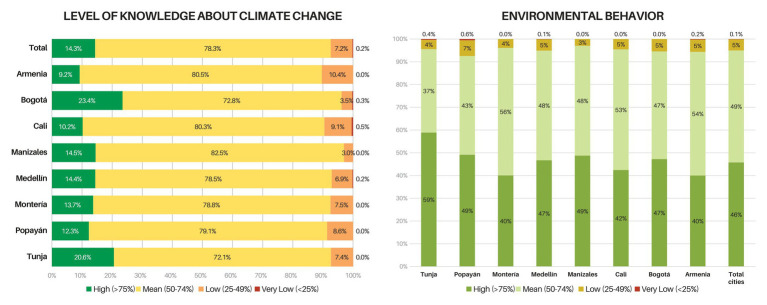
Knowledge about climate change (left) and pro-environmental behaviors (right) by cities in Colombia.

An index was calculated for each of the modules that made up the questionnaire. These indices were obtained by granting five points for answers that were (a) in accordance with the dimension *CC knowledge* or (b) consistent with the dimensions *environmental attitude (NEP)*, *self-efficacy*, *trust in sources of environmental information* and *PEBs*. This score decreases as the selection moves away from the desired response. However, there was no discount if the answer was wrong. In this way, maximum and minimum scores were established, and intervals were created with the aim of classifying all the variables within the established ranges (Figures 2, 3). In the following section, the mean values and the observed deviations for each variable are presented using as reference the values presented in Table 2.

The effects of sociodemographic variables on the reference dimensions were analyzed through tests of mean differences. As a first measure, the normality of the dimensions was analyzed through the Shapiro-Wilk test. Next, a Student’s *t*-test, ANOVA and a *Kruskal-Wallis* one-way ANOVA were conducted. For the latter, *post-hoc* tests were also carried out, such as *Tukey* and *post-hoc Kruskal-Wallis Dunn*, in order to identify which group showed the largest differences.

Table 1 also shows statistical differences between both countries for the sociodemographic variables. Section “Exploratory analysis Colombia” highlights significant differences among the two countries regarding the analyzed dimensions.

#### Regression Model: Ordinary Least Squares (OLS) and Principal Component Analysis (PCA)

Pro-environmental Behaviors (PEBs) were evaluated through the OLS method. As a first measure, the variables were standardized and five *z-score* indices were created (Eq. 1), which were defined as the weighted average of the *z-score* of their variables, following the methodology proposed by Kling and Liebman (2004) and Kling et al. (2007). Next, the following regression was made:

(1) $$y_{ic}=\alpha_{ic}+\beta \times Knowledge_{ic}+X_{ic}^{\prime }+\varepsilon_{ic}$$

Where $y_{ic}\;$is the result of interest (*Pro-environmental Behaviors*), *i* and *c* are identifiers for the individuals and the country. $X_{ic}^{\prime }\;$is the vector of sociodemographic and educational characteristics, and $\varepsilon_{ic}$ is the standard error. *β* is the coefficient that measures the effect of knowledge about CC on the PEBs of students. In the absence of identifiable exogenous shocks or other means to establish causality, estimates should be interpreted as (partial) correlations.

As a way to mitigate omitted variable biases, the variation of the β coefficient was observed when adding the covariates to the initial regression. Five different specifications were used in two phases. First, a regression of the result of interest on knowledge. Second, the inclusion of the sociodemographic characteristics of the individuals. Third, in addition to the previous models, the educational characteristics of the students were included. Fourth, in the second phase, a regression of student behavior was performed in all *z-score* indices. Fifth, all the indices and sociodemographic characteristics of the students as well as the educational variables were included in the model (Eq. 2):

(2) $$\begin{array}{l} y_{ic}=\alpha +\beta_{1}\times Knowledge+\beta_{2}\times Efficacy+\\ \;\;\;\;\;\;\;\;\;\;\;\;\;\;\beta_{3}\times Trust+\beta_{4}\times NEP+X\prime_{ic}\gamma +\varepsilon \end{array}$$

Here, *α* is the constant parameter and β_1_, β_2_, β_3_, and β_4_ are the coefficients for each of the *z-scores*. γ is the vector of coefficients for the matrix of sociodemographic and educational variables, and *ε* is the standard error of the model. The PCA method was used as an additional robustness test. This statistical procedure uses an orthogonal transformation to convert a set of observations of possibly correlated variables into a set of values of variables that are not linearly correlated. Thus, the same indices used in the OLS method were recreated with the PCA and variables were regressed on the PCA indices.

## Results

### Exploratory Analysis Nicaragua

The majority of the population (71.6%) possess moderate knowledge about CC (x̅ = 51.5; *σ* = 9.1) while only a small portion of the sample possess high CC knowledge (16.5%). As shown in Figure 2, the values obtained are slightly higher for the country’s capital, Managua, which, in turn, also has the lowest proportion of the population unaware of the causes and effects of CC. Although the mean differences in knowledge scores were low, they were significant between Managua and León (*p* < 0.01) and Managua and Matagalpa (*p* < 0.01). Men possess significantly higher CC knowledge (*p* < 0.01), and no significant differences were found for both income and education level.

Regarding the field of study, engineering students show better knowledge than those studying agricultural sciences, law, economics, pedagogy, agricultural sciences, and basic sciences (*p* < 0.05). For their part, agricultural science students present better knowledge than students from administrative sciences, basic sciences and humanities (*p* < 0.001).

Regarding CC knowledge, data reveal that the vast majority of students are aware of the responsibility of humans in global warming, and consider that individual actions can have an influence on global warming. Despite this awareness, important gaps were found. The majority of students consider that nuclear energy contributes to CC (false), that global warming does not affect agricultural activities such as agriculture and fishing (false), and that the industry sector produces the largest amount of GHG emissions (false). About half of the sample is unaware of the importance of clouds and water vapor in the atmosphere – in fact, the majority of the sample affirmed that without clouds, the earth would not be in danger (false). Despite this, a correct understanding was observed of the problems that ultraviolet radiation can cause to people’s health and of the consequences of ozone depletion (Appendix A).

Regarding self-efficacy, students are, on average, akin to 76.3% of the items (x̅ = 15.4; *σ* = 3.4), corresponding to a high level of affiliation with a perceived importance of individual actions in the environment. No significant differences on self-efficacy were found for any of the sociodemographic variables. Responses of younger people show that they are less prone to carry out active and participatory processes in mitigation and adaptation strategies to CC. In contrast, 88.8% of the surveyed population expressed high levels of awareness about the impact of human actions on CC (Appendix B).

In contrast, the results of trust in the institutions that provide environmental information were ambivalent. Although the total trust level was above 50%, this variable was not concentrated in any extreme (x̅ = 19.5; *σ* = 4.66). With a shared perspective between both genders, the national government presents the lowest trust levels. Students have more trust in NGOs, educational institutions, and the scientific community. The latter exhibits the highest trust levels (Appendix C).

As shown in Figure 2, the students’ scores on PEBs are mostly favorable (x̅ = 47.7; *σ* = 9.8). Engineering students show better PEBs than those studying humanities, administrative sciences, education, and pedagogy (*p* < 0.05). Students from humanities on the other hand, have lower PEBs levels than health and agricultural sciences students (*p* < 0.05). Several discrepancies were identified regarding the students’ real behavior: Household waste separation receives a low qualification across the whole sample. Likewise, about half of the sample prefers to use private instead of public transport. It is worth highlighting that most people tend to pay attention to savings in water and electricity consumption and decide to adopt practices that contribute to preventing the wastage of these resources (Appendix D).

The surveyed population showed affinity with ecological premises of the NEP scale. On average, 78.2% of the population respond in accordance with the overall statements (x̅ = 31.1; *σ* = 6). No significant differences were observed when testing for gender. By field of study, engineering students show better behavior for this variable than students from humanities, administrative sciences, pedagogy, and agricultural sciences (*p* < 0.05). The latter, for their part, perform better than students from administrative sciences and pedagogy, but worse than those from health sciences (*p* < 0.05). When analyzing this dimension by cities, Managua showed the best results, with significant differences to Matagalpa (*p* < 0.01) and León (*p* < 0.05). It was evidenced that students assign importance to building a better balance between humans and nature. However, affinity with the statement “*The ultimate goal of plants and animals must be to serve the needs of the population*”, presented an important variability in the distribution of the degree of affinity, and it is observed that the response rate decreases drastically compared to the previous questions (Appendix E).

### Exploratory Analysis Colombia

In general, it was observed that students from cities possess middle to high knowledge regarding CC (x̅ = 51.6; *σ* =7.8). Figure 3 shows that the majority of the student population (78.3%) is located within the intermediate knowledge and to a lesser extent within the high knowledge levels (14.3%). Regarding gender, the Colombian students show similar results as their Nicaraguan peers. However, for Colombia the differences are significant: men possess higher CC knowledge (*p* < 0.01), while women show better environmental behavior (*p* < 0.01), greater self-efficacy (*p* < 0.01), and better environmental attitude (*p* < 0.01). Regarding CC knowledge, there were no significant differences found between Colombian and Nicaraguan students. However, at the country level, significant differences were observed: engineering students present a higher knowledge level than those studying humanities, economics, basic sciences, administrative sciences, communication, humanities, and agricultural sciences (*p* < 0.05).

Bogotá (the capital city) has the most informed student population (x̅ = 54.2; *σ* =7.5), followed closely by Tunja (x̅ = 53; *σ* =8), an intermediate city close to Bogotá. Armenia, on the other hand, has the least informed student population (x̅ = 50.2; σ =7.5). The latter and Bogotá stand out for presenting more significant differences compared to the other cities of this sample. Though students shows a high understanding of the impact of global warming on agriculture and fishing, they present a wrong understanding of the contribution of nuclear energy and the industrial sector to CC. Students also ignore the relationship between ozone and ultraviolet radiation, but are aware of the effects that the latter has on people’s health (Appendix A). There were no significant mean differences observed for the effects of the academic cycle and socioeconomic strata on CC knowledge.

Compared to the results for Nicaragua, self-efficacy was significantly higher in Colombia (x̅ = 16.3; σ = 2.9; *p* < 0.01), especially in terms of individual actions to reduce global warming and CC. Although in Colombia both genders show a high degree of awareness about the impact of human actions on the environment, in all items women presented a greater degree of affinity toward the statements (Appendix B).

With regard to trust in institutions that provide environmental information, the Nicaraguan students show significantly higher levels than their Colombian peers (*p* < 0.001). Although positive responses were observed for Colombia, the means are mainly concentrated within intermediate ranges (x̅ = 19.2; σ = 4.2). Perceptions about the institutions were widely divergent. On average, students rather trust the scientific community, educational institutions, and NGOs, but are reluctant to the information offered by the government (Appendix C).

The results for the PEB dimension show that Colombian students have a significantly better behavior than their Nicaraguan peers (*p* < 0.001). This dimension is located in the upper part of the mean range (x̅ = 50.6; σ = 9). An interesting aspect is that economics students present the lowest levels of environmental behaviors compared to the other fields of study (*p* < 0.05), while the behavior of the means of the other programs was much more stable. A slight relation among PEBs per city and CC knowledge can be observed (Figure 3). Tunja stands out because it ranks second in the proportion of students with more CC knowledge and first in PEBs. In fact, it is the only city with significant differences. No significant differences were found for this dimension when analyzing socioeconomic strata. Similarities were observed with the Nicaraguan sample: students pay special attention to avoid wastage of electricity and water, but the proportion drops drastically when it comes to household waste separation (Appendix D). Environmental attitude presents the highest score among the Colombian students, who particularly display a high level of affinity with the importance of building a better balance between humans and nature (x̅ = 33; σ = 5.2). When compared with Nicaragua, this dimension was significantly higher among Colombian students (*p* < 0.001). For this variable, humanities students display better behavior than those studying engineering, economics, administrative sciences, communication, pedagogy, basic sciences, and agricultural sciences (*p* < 0.05). There is also a better behavior among law students over communication and pedagogy (*p* < 0.05), economics students over pedagogy (*p* < 0.05), and basic sciences over communication (*p* < 0.05). On average, the attitude’s score increases as the academic cycle progresses. When analyzing this variable by socioeconomic strata, the means of the attitudes show a uniform behavior. Significant differences among strata can be observed, but these differences do not have a definite pattern, or in other words, it is not possible to state whether as the strata increases there are better attitudes or vice versa. Cali stands out as the city with the highest mean behavior for this dimension (x̅ = 34.3; σ = 5.2) and presents significant differences with all other cities of this sample (*p* < 0.01). Unlike in Nicaragua, the Colombian students’ responses on the usefulness of plants and animals for the satisfaction of human needs show greater homogeneity in their distribution, mostly rejecting the statement. Likewise, it is observed that students are aware of the human-induced impact over natural resources (Appendix E).

### Regression Model: OLS and PCA

According to the Brush Pagan test, the assumption of homoscedasticity in the residuals is rejected. Therefore, robust standard errors were included in the models. Multicollinearity problems are ruled out in the variables according to the correlation matrix. Since there were no variables that could be affected by endogeneity, no control tests were performed. Assumption of normality in the residuals was verified.

Ordinary Least Squares (OLS) regressions were estimated for the integrated environmental behavior index. Tables 3 and 4 show the results of the OLS model with the *z-score* indicators for Nicaragua and Colombia, while Tables 5 and 6 show the results of the PCA. For both cases, the first column shows the result of the first regression, which measures the relationships between CC knowledge and PEBs without including control variables. The second column shows the relationships between PEBs and CC knowledge, adding sociodemographic variables, while the third column shows the relationship between the dependent variable and CC knowledge, controlling both the socioeconomic and educational variables. Finally, the fourth and fifth columns include all the indicators for the different dimensions of the regression, i.e., self-efficacy, trust in the institutions that provide environmental information and NEP.

**Table 3:** Results of the OLS model for Nicaragua.

	(1)	(2)	(3)	(4)	(5)
CC Knowledge	0.538^***^	0.561^***^	0.563^***^	0.113^***^	0.119^***^
	(0.035)	(0.041)	(0.043)	(0.032)	(0.039)
Self-efficacy				0.160^***^	0.162^***^
				(0.020)	(0.023)
Trust				0.140^***^	0.126^***^
				(0.016)	(0.019)
NEP				0.235^***^	0.249^***^
				(0.023)	(0.026)
Observations	2,312	1872	1713	2,253	1,677
R^2 adjusted	0.152	0.161	0.172	0.353	0.370
Socio-economic controls	No	Yes	Yes	No	Yes
Education controls	No	No	Yes	No	Yes

*** *p* < 0.01.

**Table 4:** Results of the OLS model for Colombia.

Dimension	(1)	(2)	(3)	(4)	(5)
CC Knowledge	0.383^***^	0.404^***^	0.408^***^	0.084^***^	0.097^***^
	(0.030)	(0.031)	(0.031)	(0.022)	(0.023)
Self-efficacy				0.229^***^	0.211^***^
				(0.013)	(0.012)
Trust				0.065^***^	0.069^***^
				(0.011)	(0.011)
NEP				0.180^***^	0.200^***^
				(0.014)	(0.014)
Observations	4,764	4,563	4,460	4,744	4,442
R^2 ajusted	0.070	0.111	0.128	0.271	0.320
Socio-economic controls	No	Yes	Yes	No	Yes
Education controls	No	No	Yes	No	Yes

*** *p* < 0.01.

**Table 5:** Results PCA Nicaragua.

Dimension	(1)	(2)	(3)	(4)	(5)
CC Knowledge	0.455^***^	0.494^***^	0.475^***^	0.087^*^	0.103^*^
	(0.045)	(0.050)	(0.053)	(0.048)	(0.058)
Self-efficacy				0.614^***^	0.621^***^
				(0.145)	(0.162)
Trust				0.526^***^	0.458^***^
				(0.115)	(0.137)
NEP				1.038^***^	1.181^***^
				(0.176)	(0.202)
Observations	736	625	583	730	579
R^2 adjusted	0.219	0.236	0.236	0.378	0.383
Socio-economic controls	No	Yes	Yes	No	Yes
Education controls	No	No	Yes	No	Yes

* *p* < 0.10;

*** *p* < 0.01.

**Table 6:** Results PCA Colombia.

Dimension	(1)	(2)	(3)	(4)	(5)
CC Knowledge	0.434^***^	0.447^***^	0.466^***^	0.120^***^	0.140^***^
	(0.037)	(0.039)	(0.040)	(0.035)	(0.036)
Self-efficacy				0.950^***^	0.901^***^
				(0.099)	(0.095)
Trust				0.306^***^	0.329^***^
				(0.080)	(0.079)
NEP				0.527^***^	0.578^***^
				(0.106)	(0.104)
Observations	1,216	1,174	1,148	1,213	1,145
R^2 adjusted	0.183	0.218	0.237	0.332	0.378
Socio-economic controls	No	Yes	Yes	No	Yes
Education Controls	No	No	Yes	No	Yes

*** *p* < 0.01.

The results from the OLS models in both countries show that the dimensions of self-efficacy, trust in information sources and NEP account for nearly 20% of the variation in the explanatory power of the model as displayed by its adjusted R-squared, with a slight reduction of the percentage of variation explained from these dimensions in the models employing PCA. On the other hand, the sociodemographic and educational controls account for 2% or less of the variation in the model for Nicaragua and 5% for Colombia, respectively.

Adequate CC knowledge is associated with better PEBs, both in Colombia and in Nicaragua, although with greater magnitude in the latter. The coefficients are statistically significant at the 1% level in all specifications. In particular, the inclusion of sociodemographic and educational control variables does not determine relevant changes in the correlation between the variables. While the coefficient decreases when adding other indexes of the survey to the regression, it is suggested that the correlation between PEBs and attitudes is explained mostly by self-efficacy, trust in the institutions that provide environmental information, and NEP.

In both countries, both NEP and self-efficacy are more correlated with PEBs than CC knowledge itself. In Nicaragua, the NEP coefficient indicates that for each increment of a standard variation, the behavior changes 0.25 standard deviations in the same direction; an increase in affinity with the NEP indicator represents a greater increase in PEBs compared to the other dimensions analyzed. The same happens in Colombia, but with self-efficacy. For each variation of one standard deviation, PEBs increases by 0.21 standard deviations.

The consistency of the results was evaluated through a PCA (Tables 5 and 6). When all the variables are included, the explanatory factor measured by the R-squared indicator is higher, which indicates a clear correlation between the behavior and the variables analyzed. Even at a disaggregated level, results were similar, meaning that significant results are not due to the aggregation of the variables in each index. In fact, the knowledge index for the survey questions has been decomposed and the correlation between each *z-score* has been estimated (Annex 6 and 7), showing that most of the coefficients are positive and statistically significant at 1%, with the exception of the outliers mentioned in the previous subsection.

For both countries, statements with negative coefficients were identified. In Colombia and Nicaragua, the statement “*the industry is the sector that produces the highest level of GHG emissions*” presented this effect. The same was found in Colombia with the statement “*the high amounts of ozone in the atmosphere increase the ultraviolet radiation on the surface of the earth*” and in Nicaragua with the statements “*Nicaragua is one of the main producers of GHG*” and “*the use of renewable energy can increase global warming*”.

Although the results indicate patterns, they might be biased due to omitted variables. Given the cross-sectional nature of the data used, it is not possible to identify the causal effect of CC knowledge, environmental attitudes, or trust in the institutions that provide environmental information on PEBs. However, the data allow controlling for the sociodemographic characteristics and educational level, and confirm the meaning and orientation of the proposed estimates.

## Discussion

Our results can contribute to policy formulation and indicate the direction of future research in various ways. First, results show that the R-squared is very similar between countries with considerable similarities but also important cultural and social differences (38%). This reveals that the measured psychological and socioeconomic factors have similar effects on PEBs across two cultures, but also that there is still a need to identify other dimensions or factors that explain the remaining variability of the PEBs and if these too are consistent.

Against our initial expectations, the results show that while the sociodemographic and educational variables are significant factors for explaining PEBs, their effect was relatively small, compared to the included dimensions. This highlights the strength of idiosyncratic and cognitive factors for explaining PEBs. A possible hypothesis suggests that a “common culture” prevails in students not related to university education, but rather explained by informal communication processes that occur in the social environment (Meira-Cartea et al., 2018). Thus, for example, widely disseminated knowledge, such as the effects of carbon dioxide in the atmosphere or the importance of the ozone layer, seem to be widely recognized, but the characterization of knowledge becomes more diffuse when trying to clarify the role of chlorofluorocarbons in the mitigation of climate change. Furthermore, this generalized knowledge denotes certain mistakes, such as when trying to clarify the contributions of nuclear energy (Appendix A). In accordance with Meira-Cartea et al. (2018), the prevalence of this general culture questions the relevance of the training received in its ability to influence the scientific and social representations of students, in this case, from both countries.

Beyond this, it is important to emphasize the need to conduct further research to identify additional sources of variation. Some studies suggest that a greater degree of explanation can be achieved by taking into account additional internal dimensions, external forces, and contextual factors. These include e.g., physical infrastructure, technical facilities, availability of products and their characteristics (Steg and Vlek, 2009), environmental policies, financial strategies (Bertoldi, 2017), social norms and the influence of the social nuclei of an individual, and the duration of and adaptation to a technology (Truelove and Gillis, 2018; Li et al., 2019). Recently, Truelove and Gillis (2018) revealed a new explanatory dimension: CC impacts on health and safety, which is of major relevance in developing countries since they are the most vulnerable to CC. Other dimensions with explanatory potential are those that combine internal and external forces (psychosocial constructs), understood as the interactions or socialization of a person with the physical world, and can stimulate the occurrence of behavior or sustainable actions. Within this construct stands out not only the fact that socio-environmental actions depend on environmental identity, altruism or the sense of equity, but also the analysis of emotions (e.g., the appreciation of nature or feelings of resentment for ecological deterioration) (Pérez Ibarra et al., 2020).

Next, based on the psychological factors included in this research, our results allow providing recommendations for the design of policies and other instruments intended to modify student behavior toward a more sustainable direction. Although CC knowledge and trust in institutions showed significant correlations with PEBs, attitudes and self-efficacy exhibit a much stronger relationship, which is why it could be fruitful to focus strategies on these dimensions. Following Spence and Pidgeon (2010), emphasizing on the potential negative impacts of not carrying out actions, abandoning alarmist paradigms, and generating more binding strategies (combining personal and collective solutions) – arise as directive vectors, which have been proven useful techniques for students (Parant et al., 2016). Nevertheless, transmission of motivational communication should be carried out carefully, as people may not respond adequately. Students in Colombia and Nicaragua (contrary to the results presented by Vignola et al. (2013) for the case of Costa Rica) do not have sufficient trust in the institutions that generate such communications, particularly in governmental sources. Thus, actions addressed at increasing trust of public sources while better leveraging on the credibility of NGOs and the scientific community in the communication strategies for adaptation and mitigation, may provide better results.

The present study did not consider the impact of economic incentives and disincentives on PEBs, which could be an additional explanatory factor. However, it was observed that students weigh the effort or the costs related to certain PEBs. This allows establishing a clear differentiation between low-cost behaviors and mobility behaviors, as described by Tobler et al. (2012b). In both countries, commitment with certain environmental actions varied according to their rigidity. While closing the water tap and turning off the light or the fan when not in use are commonly applied actions among the studied population, other, more determinant actions, like traveling less, recycling, or using the bicycle instead of a car are less common. These results are in line with several other studies, such as Tobler et al. (2012b), Steinhorst et al. (2015) or Truelove and Gillis (2018), and can be explained not only from the psychological dimension but also from an overlapping economic dimension. Although the social benefit of a given environmental action is greater than the individual cost, the agent may prefer not to apply it given his or her own assessment of the personal effort or cost involved. Truelove and Gillis (2018) found that for so-called *laypeople* (people who are not experts in PEBs), the monetary costs of carrying out a certain behavior outweighs the frequency a behavior needs to be carried out. However, several other studies (e.g., Delmas et al., 2013; Schwartz et al., 2015; Steinhorst et al., 2015) show that monetary incentives are not a good standalone solution and might even be counterproductive if they form the sole basis of environmental behavior campaigns. It is much more useful to take into account the psychological, social, and civic stimuli of conservation, especially when it comes to a population group such as university students. While monetary framing might be efficient for one particular environmental action/behavior, environmental framing goes beyond that and can motivate additional non-targeted actions or behaviors (Steinhorst et al., 2015). Thus, monetary incentives should rather be considered a complementary element in pro-environmental campaigns. This is also consistent with findings from Gifford and Comeau (2011) who observed that young people participate to a lesser extent in mitigation actions and thus should be a target of motivational strategies.

Finally, the fact that in many cases no significant differences were found could be a result of the measurement scale, which was used to provide continuity. Knowledge about CC has multiple evaluation dimensions and not all of them have the same effect on attitudes and/or behaviors toward mitigation. Given that the knowledge dimension featured here was not only focused on the causes but also on the manifestations of CC, important information might have been lost in the aim of establishing a stronger relationship. It is important to note that this situation does not imply that the used scale has measured knowledge incorrectly. On the contrary, Shi et al. (2016) recognize that CC is a complex phenomenon, which results from a function of multiple causes and presents various characteristics and consequences. Therefore, it is important to measure the perceptions of CC transversely.

## Study Limitations

The methodology used for this study assumes that students accurately reflect and report on their behaviors. However, there is no certainty on their actual PEBs. In order to examine the factors explaining actual behavior of a certain population, different approaches would have been necessary. Given the large sample size, time, and financial limitations, it was not possible to adopt such approaches in the methodology. This indicates that there may be a systematic difference between what students say they do and what they actually do, which is not captured by the selected variables. The literature is ambiguous regarding this issue. Corral-Verdugo and Figueredo (1999) and Kormos and Gifford (2014) suggest that results based on stated information may have high levels of validity in terms of predicting real behavior. Other researchers, such as Fuj et al. (1985) and Sheeran (2002), argue that these types of measurements may have inherent distortions in the process of explaining such behavior.

In addition, by adding different dimensions, not including explicitly subjective norms and by combining both declared behaviors and intentions in the PEB dimension, this study extends and deviates from the initial framework provided by the TPB. This may hinder comparison with other models following this framework more strictly. Nevertheless, both the results from the Cronbach’s alpha and PCA show that the constructs used for measuring PEBs are internally consistent, and that the added elements provide new information to further complement the original framework.

## Conclusion

This study examined the relationships of four psychological dimensions on PEBs of higher education students from two developing countries, Colombia and Nicaragua. This investigation responds to the tendency toward decentralization of global governance in environmental issues, in which more cost-efficient strategies and policies are needed. Therefore, this work is a contribution to creating empirical evidence for multiple authorities and decision-makers and helps in assessing the capacity of the civil society to contribute to the fight against CC, and thus p rovides valuable inputs for the design of more effective and efficient environmental initiatives.

By comparing countries with different cultural contexts and political systems, this study provides strong evidence that CC knowledge, trust in sources of environmental information, self-efficacy, and environmental attitudes are important predictors of PEBs in a developing country’s population.

Our literature review revealed an increasing preponderance of the topic, with studies being conducted around the globe. In Latin America, a constant rise of such studies was observed, appearing largely in South American countries, while in the Central American region they were conducted rather sporadically. Yet, findings remain obscure. Therefore, our study contributes to expanding the body of knowledge for the region and provides a point of comparison for further research within the region and across other cultures.

Our results highlight the importance of the scientific community and activists of generating and communicating information on CC for guiding public concerns toward appropriate environmental behavior, and suggest that efforts should focus on teaching and communicating CC, emphasizing on the potential impact of private and collective action, establishing trust in institutions, and reducing an anthropocentric vision of the world. This, in turn, can help people and policymakers to better address the risks and consequences of CC, and thus gain support in the construction and implementation of effective adaptation and mitigation policies and plans.

It should be noted that the relationships found in this study are dynamic and can vary as personal values, educational processes, or exposure to CC alter. What is not in doubt is that these changes are strongly dependent on the effectiveness of public policy and its congruency with the specific realities of a target population. Thus, promoting a continuous measurement of the analyzed variables will help to improve policy design and communication strategies over time.

## Data Availability Statement

The raw data supporting the conclusions of this article will be made available by the authors, without undue reservation.

## Ethics Statement

The studies involving human participants were reviewed and approved by Internal Review Board; International Center for Tropical Agriculture; E.Sweitzer@cgiar.org. Written informed consent to participate in this study was provided by the participants’ legal guardian/next of kin. Written informed consent was obtained from the individual(s), and minor(s)’ legal guardian/next of kin, for the publication of any potentially identifiable images or data included in this article.

## Author Contributions

SB and MD: Conceptualization MD, AC, KE, MR, SS, and SB: Methodology. MD, SS, MR, KE, and AC: Formal analysis. MD, SB, and AC: Writing the original draft and review and editing. MD, SB, AC, and KE: Resources. SB: Supervision and funding acquisition. MD and SB: Project administration. All authors contributed to the article and approved the submitted version.

### Conflict of Interest

The authors declare that the research was conducted in the absence of any commercial or financial relationships that could be construed as a potential conflict of interest.

## Footnotes

^1^Socioeconomic stratification in Colombia categorizes housing units in a scale of one to six according to their physical characteristics, immediate surroundings and rural or urban context. It was implemented for assigning differential public utilities rates to different strata, enabling higher strata (five and six) to pay higher rates and subsidize the costs for lower strata (one, two and three) (Departamento Administrativo Nacional de Estadística, Colombia). Retrieved from: https://www.dane.gov.co/index.php/servicios-al-ciudadano/servicios-informacion/estratificacion-socioeconomica
