# Supplementary material for: Psychological Factors Influencing Pro-environmental Behavior in Developing Countries: Evidence From Colombian and Nicaraguan Students
Source: Front Psychol. 2020 Dec 23;11:580730. doi: 10.3389/fpsyg.2020.580730 (PMC7786311; doi:10.3389/fpsyg.2020.580730)
Supplement: Supplementary file 1 [file Table_1.docx]

**Appendices**

**Appendix A.** Frequency and mean distribution of the knowledge of Climate Change

| **Statements** | **Nicaragua** | | | | | | | | | **Colombia** | | | | | | | |
| --- | --- | --- | --- | --- | --- | --- | --- | --- | --- | --- | --- | --- | --- | --- | --- | --- | --- |
|  | **Gender** | **n** | **SD (1)** | **D (2)** | **N (3)** | **A (4)** | **SA (5)** | **Prom** | **Gender** | | **n** | **SD (1)** | **D (2)** | **N (3)** | **A (4)** | **SA (5)** | **Prom** |
| Carbon dioxide in the atmosphere contributes to the global greenhouse effect (True) | F | 943 | 4,77% | 3,29% | 15,27% | 37,75% | 38,92% | 4,03 | F | | 2263 | 2% | 2% | 7% | 31% | 58% | 4,41 |
|  | M | 1189 | 6,98% | 3,62% | 10,01% | 31,71% | 47,69% | 4,10 | M | | 2322 | 3% | 2% | 6% | 28% | 61% | 4,43 |
| Violent volcanic eruptions have effect on global climate (True) | F | 1009 | 4,36% | 8,13% | 15,26% | 46,09% | 26,16% | 3,82 | F | | 2198 | 4% | 10% | 19% | 36% | 31% | 3,80 |
|  | M | 1220 | 5,49% | 8,11% | 18,28% | 39,84% | 28,28% | 3,77 | M | | 2237 | 5% | 9% | 19% | 35% | 33% | 3,82 |
| Burning of fossil fuels, especially coal, will enhance the greenhouse effect (True) | F | 968 | 5,17% | 5,68% | 10,33% | 36,88% | 41,94% | 4,05 | F | | 2303 | 2% | 1% | 5% | 30% | 62% | 4,49 |
|  | M | 1216 | 5,84% | 3,78% | 9,54% | 30,92% | 49,92% | 4,15 | M | | 2347 | 3% | 1% | 5% | 28% | 62% | 4,46 |
| If global warming occurs, it will probably have little impact on agriculture and fishing activities in central America (False)) | F | 980 | 25,61% | 13,67% | 10,20% | 22,65% | 27,86% | 3,13 | F | | 2299 | 56% | 16% | 5% | 11% | 12% | 2,07 |
|  | M | 1215 | 28,64% | 16,05% | 9,88% | 20,25% | 25,19% | 2,97 | M | | 2324 | 51% | 17% | 7% | 12% | 13% | 2,19 |
| Nuclear energy production contributes to global warming intensification (False) | F | 939 | 4,37% | 6,18% | 18,96% | 41,96% | 28,54% | 3,84 | F | | 2086 | 5% | 6% | 19% | 34% | 36% | 3,90 |
|  | M | 1188 | 7,74% | 7,07% | 21,46% | 31,99% | 31,73% | 3,73 | M | | 2118 | 9% | 12% | 23% | 29% | 27% | 3,53 |
| Without clouds and water vapor in the atmosphere, the earth is endangered (True) | F | 913 | 18,29% | 21,58% | 22,45% | 24,10% | 13,58% | 2,93 | F | | 1924 | 28% | 22% | 21% | 16% | 13% | 2,64 |
|  | M | 1153 | 25,24% | 23,24% | 20,99% | 15,87% | 14,66% | 2,71 | M | | 2084 | 36% | 21% | 16% | 14% | 13% | 2,47 |
| Without the ozone layer in the atmosphere, life on earth is endangered (True) | F | 1007 | 6,16% | 2,09% | 3,57% | 19,66% | 68,52% | 4,42 | F | | 2309 | 3% | 1% | 2% | 15% | 80% | 4,69 |
|  | M | 1230 | 6,59% | 2,44% | 2,76% | 15,85% | 72,36% | 4,45 | M | | 2319 | 4% | 1% | 2% | 13% | 80% | 4,63 |
| Large amounts of ozone gas in the atmosphere increases ultraviolet radiation on the earth´s surface (True) | F | 892 | 16,14% | 12,67% | 19,39% | 27,24% | 24,55% | 3,31 | F | | 1926 | 30% | 18% | 16% | 17% | 19% | 2,77 |
|  | M | 1169 | 24,21% | 18,99% | 20,02% | 17,88% | 18,91% | 2,88 | M | | 2056 | 41% | 18% | 14% | 14% | 13% | 2,41 |
| Chlorofluorocarbons (CFCs) are the most serious threat to the ozone layer (True) | F | 713 | 3,79% | 6,31% | 37,45% | 31,42% | 21,04% | 3,60 | F | | 1489 | 2% | 4% | 26% | 34% | 34% | 3,92 |
|  | M | 948 | 4,64% | 5,27% | 36,08% | 28,48% | 25,53% | 3,65 | M | | 1584 | 3% | 5% | 27% | 31% | 35% | 3,90 |
| The reduction of the ozone layer contributes to the increase of global warming (True) | F | 993 | 4,73% | 2,62% | 6,04% | 28,40% | 58,21% | 4,33 | F | | 2275 | 2% | 2% | 4% | 22% | 69% | 4,54 |
|  | M | 1219 | 5,58% | 2,54% | 5,09% | 26,83% | 59,97% | 4,33 | M | | 2306 | 3% | 3% | 5% | 22% | 66% | 4,45 |
| The felling of tropical forests intensifies the global greenhouse effect (True) | F | 952 | 3,89% | 3,68% | 8,51% | 29,83% | 54,10% | 4,27 | F | | 2291 | 2% | 2% | 6% | 23% | 67% | 4,51 |
|  | M | 1205 | 6,14% | 3,82% | 6,80% | 26,47% | 56,68% | 4,28 | M | | 2300 | 2% | 3% | 5% | 26% | 64% | 4,46 |
| Nicaragua/Colombia is one of the world's largest producer of greenhouse gases (False) | F | 847 | 20,31% | 26,92% | 31,05% | 14,05% | 7,67% | 2,62 | F | | 1893 | 18% | 32% | 35% | 11% | 5% | 2,55 |
|  | M | 1146 | 30,45% | 27,92% | 25,74% | 10,12% | 5,76% | 2,33 | M | | 2045 | 26% | 32% | 28% | 9% | 4% | 2,33 |
| With the use of renewable energy (solar and wind), global warming will intensify (False) | F | 967 | 37,95% | 26,99% | 14,27% | 12,82% | 7,96% | 2,26 | F | | 2271 | 64% | 23% | 6% | 4% | 4% | 1,60 |
|  | M | 1222 | 50,00% | 22,83% | 11,70% | 8,59% | 6,87% | 2,00 | M | | 2327 | 69% | 17% | 6% | 3% | 4% | 1,55 |
| Greater exposure to ultraviolet radiation generally increases the risk of contracting skin cancer and developing cataracts of the eye (True) | F | 1028 | 5,64% | 1,26% | 4,18% | 25,68% | 63,13% | 4,40 | F | | 2310 | 2% | 1% | 4% | 23% | 71% | 4,61 |
|  | M | 1242 | 5,07% | 2,25% | 3,46% | 29,15% | 60,06% | 4,37 | M | | 2314 | 2% | 2% | 3% | 23% | 70% | 4,56 |
| Scientifics have found sufficient evidence to affirm that in Nicaragua atmospheric ozone levels are being reduced (False) | F | 751 | 7,06% | 12,12% | 47,27% | 23,04% | 10,52% | 3,18 | F | | 1413 | 6% | 14% | 44% | 25% | 12% | 3,22 |
|  | M | 1032 | 8,24% | 12,21% | 46,90% | 23,06% | 9,59% | 3,14 | M | | 1490 | 7% | 11% | 46% | 25% | 10% | 3,19 |
| Industry is the sector that produces the most greenhouse gas emissions worldwide (False) | F | 924 | 3,46% | 4,65% | 15,26% | 34,52% | 42,10% | 4,07 | F | | 2251 | 2% | 4% | 11% | 35% | 49% | 4,24 |
|  | M | 1180 | 5,34% | 4,49% | 12,97% | 32,37% | 44,83% | 4,07 | M | | 2264 | 4% | 5% | 11% | 32% | 48% | 4,15 |
| **Note:** F=Female, M=Male, SD= Strongly Disagree, D=Disagree, N= Neutral, A= Agree SA= Strongly Agree | | | | | | | | | | | | | | | | | |

**Appendix B.** Frequency and mean distribution of self-efficacy in Nicaragua and Colombia

| **Statements** | **Nicaragua** | | | | | | | | | **Colombia** | | | | | | | |
| --- | --- | --- | --- | --- | --- | --- | --- | --- | --- | --- | --- | --- | --- | --- | --- | --- | --- |
|  | **Gender** | **n** | **SD (1)** | **D (2)** | **N (3)** | **A (4)** | **SA (5)** | **Prom** | **Gender** | | **n** | **SD (1)** | **D (2)** | **N (3)** | **A (4)** | **SA (5)** | **Prom** |
| I consider that individual actions have an influence on global warming and climate change | F | 1020 | 5,20% | 3,92% | 10,78% | 31,47% | 48,53% | 4,15 | F | | 2340 | 1% | 2% | 8% | 28% | 60% | 4,44 |
|  | M | 1239 | 6,05% | 4,52% | 14,04% | 34,38% | 40,92% | 4,00 | M | | 2347 | 3% | 3% | 11% | 32% | 51% | 4,26 |
| My actions to reduce the effects of global warming and climate change will encourage others to reduce their relevant effects in the community | F | 1015 | 4,14% | 6,01% | 22,86% | 34,68% | 32,32% | 3,85 | F | | 2334 | 2% | 6% | 23% | 35% | 34% | 3,93 |
|  | M | 1235 | 6,56% | 7,85% | 22,02% | 33,44% | 30,04% | 3,73 | M | | 2344 | 4% | 7% | 22% | 34% | 32% | 3,82 |
| In my opinion, humans are the main responsibles of global warming and climate change | F | 1018 | 4,32% | 1,96% | 4,62% | 20,24% | 68,86% | 4,47 | F | | 2339 | 2% | 2% | 5% | 21% | 71% | 4,58 |
|  | M | 1241 | 4,19% | 2,34% | 4,67% | 17,73% | 70,91% | 4,50 | M | | 2351 | 3% | 3% | 6% | 20% | 68% | 4,48 |
| I try create awareness of the risk of global warming | F | 964 | 5,39% | 5,08% | 26,24% | 37,66% | 25,62% | 3,73 | F | | 2304 | 3% | 5% | 23% | 37% | 33% | 3,92 |
|  | M | 1192 | 6,04% | 5,87% | 29,70% | 35,82% | 22,48% | 3,63 | M | | 2315 | 5% | 7% | 27% | 35% | 26% | 3,69 |
| **Note:** F=Female, M=Male, SD= Strongly Disagree, D=Disagree, N= Neutral, A= Agree SA= Strongly Agree | | | | | | | | | | | | | | | | | |

**Appendix C.** Frequency and mean distribution of trust in informational resources in Nicaragua and Colombia

| **Statements** | **Nicaragua** | | | | | | | | **Colombia** | | | | | | | |
| --- | --- | --- | --- | --- | --- | --- | --- | --- | --- | --- | --- | --- | --- | --- | --- | --- |
|  | **Gender** | **n** | **SD (1)** | **D (2)** | **N (3)** | **A (4)** | **SA (5)** | **Prom** | **Gender** | **n** | **SD (1)** | **D (2)** | **N (3)** | **A (4)** | **SA (5)** | **Prom** |
| I trust the information the government provides on climate change | F | 988 | 14,37% | 21,05% | 33,91% | 23,18% | 7,49% | 2,88 | F | 2323 | 20% | 27% | 36% | 14% | 3% | 2,53 |
|  | M | 1221 | 17,20% | 20,15% | 29,98% | 22,85% | 9,83% | 2,88 | M | 2333 | 23% | 25% | 31% | 17% | 5% | 2,56 |
| I trust the information the non-governmental agencies provide on climate change | F | 988 | 5,36% | 9,62% | 32,49% | 35,73% | 16,80% | 3,49 | F | 2325 | 6% | 10% | 29% | 38% | 16% | 3,47 |
|  | M | 1221 | 7,86% | 9,66% | 30,30% | 35,05% | 17,12% | 3,44 | M | 2320 | 7% | 11% | 27% | 37% | 19% | 3,49 |
| I trust the information provided for scientific community on climate change | F | 998 | 3,71% | 6,91% | 22,14% | 41,28% | 25,95% | 3,79 | F | 2336 | 3% | 4% | 13% | 39% | 41% | 4,13 |
|  | M | 1220 | 5,25% | 5,66% | 20,08% | 41,39% | 27,62% | 3,80 | M | 2345 | 3% | 4% | 13% | 38% | 42% | 4,13 |
| I trust information provided for Nicaraguan/Colombian educational institutions on climate change | F | 993 | 5,04% | 8,86% | 31,52% | 38,77% | 15,81% | 3,51 | F | 2332 | 5% | 10% | 32% | 38% | 14% | 3,46 |
|  | M | 1230 | 7,80% | 8,70% | 28,37% | 36,26% | 18,86% | 3,50 | M | 2345 | 6% | 10% | 29% | 38% | 18% | 3,52 |
| In my opinion, the average Nicaraguan/Colombian is aware of causes and effects of global warming and climate change | F | 995 | 12,56% | 20,50% | 21,71% | 27,24% | 17,99% | 3,18 | F | 2303 | 21% | 35% | 21% | 17% | 6% | 2,52 |
|  | M | 1218 | 16,01% | 21,76% | 20,44% | 24,96% | 16,83% | 3,05 | M | 2309 | 22% | 34% | 20% | 17% | 8% | 2,54 |
| In my opinion, the groups of defense of the environment have correct information on global warming and climate change | F | 971 | 4,63% | 8,65% | 32,44% | 36,77% | 17,51% | 3,54 | F | 2218 | 3% | 8% | 34% | 40% | 15% | 3,55 |
|  | M | 1193 | 6,71% | 9,22% | 28,67% | 36,55% | 18,86% | 3,52 | M | 2232 | 4% | 9% | 32% | 38% | 18% | 3,57 |
| **Note:** F=Female, M=Male, SD= Strongly Disagree, D=Disagree, N= Neutral, A= Agree SA= Strongly Agree | | | | | | | | | | | | | | | | |

**Appendix D**. Frequency and mean distribution of perceived pro-environmental behaviors in Nicaragua and Colombia

| **Statements** | **Nicaragua** | | | | | | | | | **Colombia** | | | | | | | |
| --- | --- | --- | --- | --- | --- | --- | --- | --- | --- | --- | --- | --- | --- | --- | --- | --- | --- |
|  | **Gender** | **n** | **SD (1)** | **D (2)** | **N (3)** | **A (4)** | **SA (5)** | **Prom** | **Gender** | | **n** | **SD (1)** | **D (2)** | **N (3)** | **A (4)** | **SA (5)** | **Prom** |
| While I brush my teeth’s I do not let the water run | F | 1023 | 4,11% | 3,23% | 4,69% | 27,37% | 60,61% | 4,37 | F | | 2347 | 2% | 1% | 3% | 15% | 79% | 4,69 |
|  | M | 1243 | 5,39% | 5,07% | 7,16% | 23,73% | 58,57% | 4,25 | M | | 2351 | 2% | 2% | 5% | 18% | 73% | 4,57 |
| When I leave a room I turn off the lights | F | 1027 | 3,60% | 3,80% | 9,54% | 28,53% | 54,53% | 4,27 | F | | 2351 | 1% | 2% | 6% | 24% | 66% | 4,51 |
|  | M | 1241 | 4,75% | 4,67% | 9,19% | 28,04% | 53,26% | 4,21 | M | | 2357 | 2% | 2% | 9% | 25% | 61% | 4,41 |
| When I leave the house I turn off the fan and/or the air conditioner | F | 984 | 4,47% | 2,74% | 5,79% | 24,90% | 62,09% | 4,37 | F | | 2090 | 2% | 1% | 2% | 10% | 85% | 4,76 |
|  | M | 1191 | 3,86% | 3,36% | 8,31% | 24,35% | 60,03% | 4,34 | M | | 2135 | 3% | 1% | 4% | 14% | 78% | 4,63 |
| My fan/air conditioner is on every day for a long time | F | 941 | 32,73% | 19,77% | 19,34% | 14,35% | 13,82% | 2,57 | F | | 2041 | 70% | 12% | 7% | 5% | 7% | 1,67 |
|  | M | 1162 | 31,33% | 21,43% | 18,67% | 15,23% | 13,34% | 2,58 | M | | 2084 | 60% | 17% | 9% | 7% | 8% | 1,87 |
| I take short showers to reduce water consumption in my home | F | 1011 | 10,19% | 12,46% | 23,05% | 27,79% | 26,51% | 3,48 | F | | 2345 | 5% | 10% | 25% | 25% | 35% | 3,76 |
|  | M | 1224 | 11,85% | 11,60% | 21,73% | 26,14% | 28,59% | 3,48 | M | | 2355 | 7% | 10% | 22% | 27% | 33% | 3,69 |
| I do not waste food | F | 1012 | 7,11% | 9,09% | 21,64% | 28,36% | 33,79% | 3,73 | F | | 2346 | 3% | 7% | 20% | 33% | 37% | 3,94 |
|  | M | 1237 | 6,95% | 7,44% | 16,25% | 26,84% | 42,44% | 3,91 | M | | 2347 | 4% | 5% | 16% | 27% | 48% | 4,09 |
| If I see someone throwing garbage in the street I tell him he should not do it | F | 995 | 8,74% | 11,26% | 27,94% | 23,12% | 28,94% | 3,52 | F | | 2304 | 8% | 12% | 29% | 26% | 25% | 3,48 |
|  | M | 1214 | 12,60% | 14,66% | 26,85% | 22,82% | 23,06% | 3,29 | M | | 2321 | 13% | 14% | 33% | 22% | 18% | 3,19 |
| I turn off the TV or computer when I'm not using them | F | 1015 | 6,21% | 5,62% | 11,03% | 26,60% | 50,54% | 4,10 | F | | 2345 | 3% | 6% | 12% | 26% | 53% | 4,21 |
|  | M | 1227 | 7,74% | 6,36% | 13,20% | 26,41% | 46,21% | 3,97 | M | | 2348 | 5% | 8% | 15% | 28% | 44% | 3,98 |
| I use the same item of clothing more than once week before washing it to reduce water consumption in my home | F | 1014 | 17,16% | 13,71% | 16,86% | 23,77% | 28,50% | 3,33 | F | | 2346 | 9% | 8% | 14% | 26% | 44% | 3,89 |
|  | M | 1234 | 14,75% | 12,32% | 15,15% | 26,18% | 31,60% | 3,48 | M | | 2359 | 9% | 8% | 15% | 31% | 38% | 3,80 |
| I separate the garbage in my home | F | 1013 | 21,03% | 22,21% | 20,93% | 19,55% | 16,19% | 2,90 | F | | 2329 | 19% | 19% | 20% | 19% | 22% | 3,05 |
|  | M | 1229 | 21,48% | 20,34% | 20,91% | 20,18% | 17,01% | 2,91 | M | | 2341 | 21% | 20% | 23% | 18% | 18% | 2,91 |
| I avoid using private transportation to reduce pollution | F | 915 | 20,00% | 15,96% | 23,61% | 18,47% | 21,97% | 3,06 | F | | 2002 | 17% | 14% | 19% | 18% | 32% | 3,35 |
|  | M | 1135 | 19,91% | 14,19% | 24,85% | 17,09% | 23,96% | 3,11 | M | | 2080 | 19% | 14% | 19% | 18% | 30% | 3,24 |
| I use bicycle and/or road to avoid contamination when transporting | F | 945 | 20,74% | 14,71% | 22,86% | 18,84% | 22,86% | 3,08 | F | | 2242 | 21% | 16% | 21% | 18% | 25% | 3,11 |
|  | M | 1200 | 19,08% | 12,83% | 20,83% | 19,75% | 27,50% | 3,24 | M | | 2281 | 17% | 13% | 20% | 19% | 31% | 3,34 |
| I'm willing to travel less frequently to reduce my impact on the environment | F | 962 | 12,68% | 11,85% | 32,22% | 23,28% | 19,96% | 3,26 | F | | 2243 | 13% | 16% | 33% | 19% | 19% | 3,14 |
|  | M | 1201 | 14,57% | 13,57% | 28,23% | 23,06% | 20,57% | 3,21 | M | | 2299 | 15% | 13% | 31% | 21% | 21% | 3,18 |
| I'm willing to change my diet to reduce my impact on the environment | F | 976 | 13,22% | 9,02% | 25,82% | 26,95% | 25,00% | 3,41 | F | | 2308 | 9% | 10% | 25% | 28% | 28% | 3,57 |
|  | M | 1203 | 15,30% | 12,80% | 29,59% | 21,95% | 20,37% | 3,19 | M | | 2303 | 14% | 13% | 27% | 24% | 22% | 3,26 |
| **Note:** F=Female, M=Male, SD= Strongly Disagree, D=Disagree, N= Neutral, A= Agree SA= Strongly Agree | | | | | | | | | | | | | | | | | |

**Appendix E.** Frequency and mean distribution of the NEP scale in Nicaragua and Colombia

| **Statements** | **Nicaragua** | | | | | | | | **Colombia** | | | | | | | | |
| --- | --- | --- | --- | --- | --- | --- | --- | --- | --- | --- | --- | --- | --- | --- | --- | --- | --- |
|  | **Género** | **n** | **TED (1)** | **ED (2)** | **N (3)** | **DA (4)** | **TDA (5)** | **Prom** | | **Género** | **n** | **TED (1)** | **ED (2)** | **N (3)** | **DA (4)** | **TDA (5)** | **Prom** |
| In order to survive, humans must live in harmony with nature | F | 1029 | 3,50% | 2,04% | 5,64% | 21,28% | 67,54% | 4,47 | | F | 2345 | 1% | 2% | 6% | 24% | 67% | 4,53 |
|  | M | 1243 | 4,51% | 2,09% | 6,19% | 22,04% | 65,08% | 4,41 | | M | 2351 | 2% | 2% | 7% | 26% | 62% | 4,43 |
| Human intervention in nature often produces disastrous results | F | 1011 | 3,36% | 1,48% | 6,82% | 30,07% | 58,26% | 4,38 | | F | 2343 | 2% | 2% | 7% | 27% | 63% | 4,47 |
|  | M | 1234 | 4,21% | 3,00% | 10,29% | 28,61% | 53,81% | 4,25 | | M | 2353 | 2% | 2% | 11% | 31% | 54% | 4,32 |
| To maintain a healthy economy, it requires a developing economy where industrial growth is controlled | F | 1001 | 2,90% | 2,30% | 12,59% | 32,57% | 49,65% | 4,24 | | F | 2319 | 2% | 2% | 15% | 32% | 49% | 4,25 |
|  | M | 1217 | 4,60% | 4,11% | 15,28% | 34,02% | 41,91% | 4,05 | | M | 2321 | 3% | 4% | 15% | 35% | 43% | 4,10 |
| Humans are severely abusing the environment | F | 1024 | 3,32% | 1,56% | 4,69% | 20,61% | 69,82% | 4,52 | | F | 2340 | 2% | 1% | 2% | 15% | 80% | 4,73 |
|  | M | 1229 | 3,58% | 3,01% | 4,31% | 19,77% | 69,32% | 4,48 | | M | 2352 | 1% | 1% | 4% | 22% | 72% | 4,62 |
| The earth is like a spaceship with very limited room and resources | F | 988 | 5,87% | 5,67% | 16,50% | 29,05% | 42,91% | 3,97 | | F | 2319 | 4% | 5% | 11% | 25% | 54% | 4,19 |
|  | M | 1203 | 6,23% | 6,15% | 14,46% | 26,10% | 47,05% | 4,02 | | M | 2324 | 4% | 4% | 12% | 29% | 50% | 4,16 |
| The primary purpose of the creation of plants and animals is to satisfy human needs | F | 979 | 21,76% | 19,00% | 24,92% | 18,79% | 15,53% | 2,87 | | F | 2321 | 43% | 24% | 17% | 10% | 8% | 2,16 |
|  | M | 1199 | 22,35% | 17,18% | 23,69% | 19,68% | 17,10% | 2,92 | | M | 2335 | 36% | 22% | 21% | 12% | 9% | 2,38 |
| We are reaching the stage where the earth cannot meet the needs of the population anymore | F | 1005 | 3,58% | 1,99% | 9,25% | 29,45% | 55,72% | 4,32 | | F | 2340 | 3% | 3% | 7% | 27% | 61% | 4,42 |
|  | M | 1230 | 6,10% | 3,74% | 9,67% | 27,32% | 53,17% | 4,18 | | M | 2337 | 3% | 4% | 9% | 31% | 53% | 4,28 |
| The growth of societies is limited by the natural characteristics of the environment | F | 958 | 6,05% | 6,78% | 24,84% | 29,02% | 33,30% | 3,77 | | F | 2251 | 10% | 11% | 23% | 24% | 33% | 3,60 |
|  | M | 1195 | 9,37% | 7,70% | 23,51% | 28,12% | 31,30% | 3,64 | | M | 2285 | 8% | 10% | 19% | 30% | 33% | 3,70 |
| **Note:** F=Female, M=Male, SD= Strongly Disagree, D=Disagree, N= Neutral, A= Agree SA= Strongly Agree | | | | | | | | | | | | | | | | | |
